# Transcription factors interact with Arabidopsis UVR8 photoreceptor at distinct sites to COP1 and RUP proteins

**DOI:** 10.1111/tpj.71009

**Published:** 2026-07-01

**Authors:** Giovanni Giuriani, Wei Liu, Gareth I. Jenkins

**Affiliations:** ^1^ School of Molecular Biosciences, College of Medical, Veterinary and Life Sciences University of Glasgow Glasgow G12 8QQ UK

**Keywords:** light signalling, photomorphogenesis, photoreceptor, protein interactions, transcription factors, UV‐B, UVR8

## Abstract

Many responses of plants to UV‐B radiation are mediated by the UVR8 photoreceptor. UVR8 functions by interacting directly with various proteins. In particular, binding of transcription factors to UVR8 modifies their ability to regulate sets of genes involved in specific responses to UV‐B. However, very little information is available on how UVR8 binds to transcription factors, although this is crucial to understand the basis of responses to UV‐B. Here, we studied the interaction of UVR8 with four transcription factors: WRKY36, BIM1, BES1 and MYB13, using co‐immunoprecipitation assays in *Nicotiana*. We show that WRKY36 binds to a 9‐amino acid region in the UVR8 C‐terminus but can also interact with the β‐propellor core domain. BIM1 binds to a different region within the C‐terminus but there is no evidence of binding to the core domain. The VP motif in the C‐terminus required for interaction of COP1 and RUP proteins is not required for binding WRKY36 and BIM1. BES1 and MYB13 do not require interaction with the C‐terminus to bind to UVR8. We conclude that transcription factors have different modes of interaction with UVR8 and that those studied here do not interact in the same way as COP1 and RUP proteins. We suggest that the less conserved regions of the C‐terminus may facilitate interaction of UVR8 with multiple transcription factors that mediate responses to UV‐B in different plant species.

## Significance Statement

Photomorphogenic responses to UV‐B radiation are initiated by the UVR8 photoreceptor through interactions with proteins, including various transcription factors. We show that four transcription factors bind to UVR8 at different sites, which are distinct to the binding sites for COP1 and RUP proteins, and suggest that variation between species in the sequence of the UVR8 C‐terminus may facilitate interactions with regulatory proteins.

## INTRODUCTION

Exposure of plants to ultraviolet‐B radiation (UV‐B) in sunlight initiates a range of metabolic, morphological and physiological responses underpinned by differential gene expression (Chen et al., 2022; Jenkins, 2017; Podolec et al., 2021; Shi & Liu, 2021). These responses enable plants to acclimate to the ambient level of UV‐B, promoting their viability under the prevailing environmental conditions. Research, principally in Arabidopsis, has shown that many of these responses to UV‐B are initiated by the photoreceptor UV RESISTANCE LOCUS 8 (UVR8) (Brown et al., 2005; Jenkins, 2017; Podolec et al., 2021; Rizzini et al., 2011). UVR8 forms homodimers in the absence of UV‐B, whereas exposure to UV‐B or short wavelength UV‐A light causes dimers to dissociate, generating signalling‐active monomers that initiate transcriptional responses (Christie et al., 2012; Qian et al., 2020; Rai et al., 2020; Rizzini et al., 2011; Wu et al., 2012). Monomeric UVR8 is also capable of absorbing UV‐B to initiate signalling (Heilmann et al., 2016). UVR8 monomers are able to re‐dimerise, a process facilitated by REPRESSOR OF UV‐B PHOTOMORPHOGENESIS 1 (RUP1) and RUP2 proteins (Heijde & Ulm, 2013). Under prolonged UV‐B exposure, as would be found in nature, UVR8 establishes a dimer/monomer photoequilibrium, where UV‐B‐induced monomerisation is balanced by RUP‐mediated dimer formation (Findlay & Jenkins, 2016; Liao et al., 2020).

UVR8 function is dependent on interactions with other proteins (Chen et al., 2022; Liu & Jenkins, 2024; Podolec et al., 2021; Shi & Liu, 2021). First, binding of RUP proteins to UVR8 mediates dimerisation (Heijde & Ulm, 2013; Wang et al., 2023). Second, UVR8 interacts with CONSTITUTIVELY PHOTOMOROPHOGENIC 1 (COP1), which together with a SUPPRESSOR OF PHYA‐105 (SPA) protein is a substrate receptor for specific E3 ubiquitin–ligase complexes that degrade the ELONGATED HYPOCOTYL 5 (HY5) transcription factor (Favory et al., 2009; Huang et al., 2013; Wang et al., 2022). HY5, sometimes acting redundantly with the closely similar HY5 HOMOLOG (HYH), is a key positive regulator of transcriptional responses initiated by UVR8 (Brown et al., 2005; Brown & Jenkins, 2008; Favory et al., 2009). Binding of COP1 to UVR8 in the nucleus following UV‐B exposure impairs HY5 degradation, leading to activation of transcription of many UVR8‐regulated genes (Favory et al., 2009; Huang et al., 2013). In addition, the interaction of COP1 with UVR8 leads to de‐stabilisation of PHYTOCHROME INTERACTION FACTOR 5 (PIF5) transcription factor, thereby helping to suppress extension growth in UV‐B (Sharma et al., 2019). Third, UVR8 interacts directly with several transcription factors to modify expression of sets of target genes. Binding of UVR8 to WRKY DNA‐BINDING PROTEIN 36 (WRKY36) in the nucleus following UV‐B exposure prevents WRKY36 suppressing transcription of the *HY5* gene, hence promoting transcription of UVR8‐target genes regulated by HY5 (Yang et al., 2018). UVR8 also binds to BRI1‐EMS‐SUPPRESSOR 1 (BES1) in its de‐phosphorylated form and to BES1‐INTERACTING MYC‐LIKE1 (BIM1), which regulate transcription of gene targets of brassinosteroid signalling; consequently, binding of BES1 and BIM1 to UVR8 following UV‐B exposure contributes to the suppression of extension growth (Liang et al., 2018). Also, in relation to the regulation of extension growth, Zhao et al. (2023) reported an interaction between *Cucumis sativus* (cucumber) CsUVR8 and the CsPIF3 transcription factor. Interaction of UVR8 with the MYB DOMAIN PROTEIN transcription factors, MYB77, MYB73 and MYB13, modifies transcription of auxin‐regulated genes and flavonoid biosynthesis genes, with impacts on lateral root growth, cotyledon expansion and UV‐B‐stress tolerance (Qian et al., 2020; Yang et al., 2020). Furthermore, a recent study (Li et al., 2024) reports that UVR8 binds to the transcription factor TEOSINTEBRANCHED 1, cycloidea and PCF 4 (TCP4) to promote expression of the jasmonic acid biosynthesis gene LIPOXYGENASE 2 (LOX2) and enhance UV‐B‐stress tolerance. Finally, UVR8 interacts with the DNA methyltransferase DOMAINS REARRANGED METHYLTRANSFERASE 2 (DRM2) to impair DNA methylation (Jiang et al., 2021) and with LIPOXYGENASE 1 (LOX1) to promote stomatal closure (Liu et al., 2025).

Given that UVR8 is able to interact with a number of different transcription factors, it is evident that the identity of the transcriptional regulator(s) binding in a particular cell at a particular time will determine the nature of the response(s) to UV‐B (Liu & Jenkins, 2024). Hence, defining how transcription factors and other proteins bind to UVR8 and how binding is regulated is key to understanding UVR8 function in plant responses to UV‐B. Detailed information has been obtained on the sites of interaction of UVR8 with COP1 (Cloix et al., 2012; Lau et al., 2019; Rizzini et al., 2011; Wang et al., 2022; Yin et al., 2015) and RUP proteins (Cloix et al., 2012; Wang et al., 2023; Yin et al., 2015), and on the role of UVR8 phosphorylation in regulating RUP binding (Liu et al., 2024). COP1 and RUPs interact with both the disordered C27 domain in the C‐terminal region of UVR8 and the β‐propellor core domain (Cloix et al., 2012; Wang et al., 2022; Wang et al., 2023; Yin et al., 2015). Amino acids in the binding sites have been identified (Wang et al., 2022, Wang et al., 2023) and a VP motif in the C27 domain is known to be required for binding (Lau et al., 2019; Wang et al., 2022; Wang et al., 2023; Yin et al., 2015). In contrast, very little information is available on the molecular basis of UVR8 binding to transcription factors. There is evidence that some transcription factors interact with the C‐terminal region of UVR8 (Liang et al., 2018; Yang et al., 2018; Yang et al., 2020), based on experiments using either *in vitro* pull‐down, yeast two‐hybrid or interaction assays with plants. Plant‐based assays have *in vivo* relevance, but are more complex because of the presence of other plant proteins and the possibility of interference, competition or cooperative binding. The purpose of this study was to initiate analysis of the interaction sites of transcription factors with UVR8 using a plant system. The study focused on four transcription factors, WRKY36, BIM1, BES1 and MYB13. The results highlight the complexity of interactions and show that transcription factors bind UVR8 at several sites and that they interact differently to COP1 and RUP proteins.

## RESULTS

To examine the interactions of UVR8 with transcription factors in plants, we used a co‐immunoprecipitation assay in which GFP‐tagged wild‐type UVR8 and selected mutants were expressed in *Nicotiana benthamiana* leaves along with a GST‐tagged fusion of the relevant transcription factor. Expression of the fusions was demonstrated in the ‘Input’ protein samples obtained from leaf extracts. GFP‐UVR8 was pulled down using GFP‐trap beads (shown by the IP sample) and the presence of the co‐immunoprecipitated transcription factor (CoIP sample) was assayed using an anti‐GST antibody. Experiments were repeated multiple times because of the variability of the assays and data are presented both as Western blots and by quantification, normalising the CoIP signal to the IP signal and adjusting for the level of Input of the transcription factor fusion. We used this assay previously to show the effect of UV‐B exposure on interaction of UVR8 with COP1 and RUP proteins (Liao et al., 2020; Liu et al., 2024).

### WRKY36 interacts with amino acids 415–423 in the C27 region of UVR8

**Figure 1:**
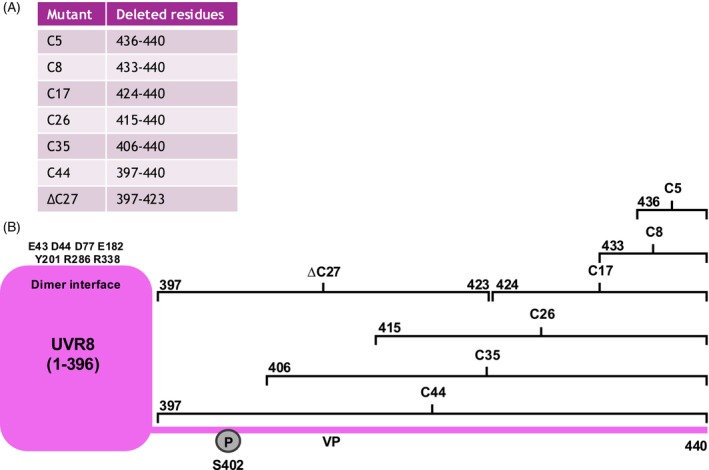
Mutants used in this work. (A) Nomenclature of the deletion mutants used in this study. ∆ is used to differentiate an internal deletion from terminal deletions. Residue numbers for the deleted amino acids are shown. (B) Diagram of the UVR8 core domain and C‐terminus. The positions of all deletion mutants within the UVR8 C‐terminus are shown and their beginning/end residues are labelled. Positions of phosphorylated S402 and the VP motif are shown. The dimer interface residues of the UVR8 core domain that were investigated are labelled.

Yang et al. (2018) reported that WRKY36 interacts with the C‐terminal region (amino acids 397–440) of UVR8 using both *in vitro* binding and bimolecular fluorescence complementation assays. To examine the interaction in *Nicotiana* CoIP assays, we initially tested a deletion of the C‐terminal region (C44; Figure 1) and an internal deletion of the C27 region that is important in interactions with RUP proteins and COP1 (ΔC27; amino acids 397–423; Figure 1). We also tested a deletion of the C‐terminal 17 amino acid region (C17; Figure 1), which is reported to inhibit interaction of C27 with COP1 (Lin et al., 2020).

**Figure 2:**
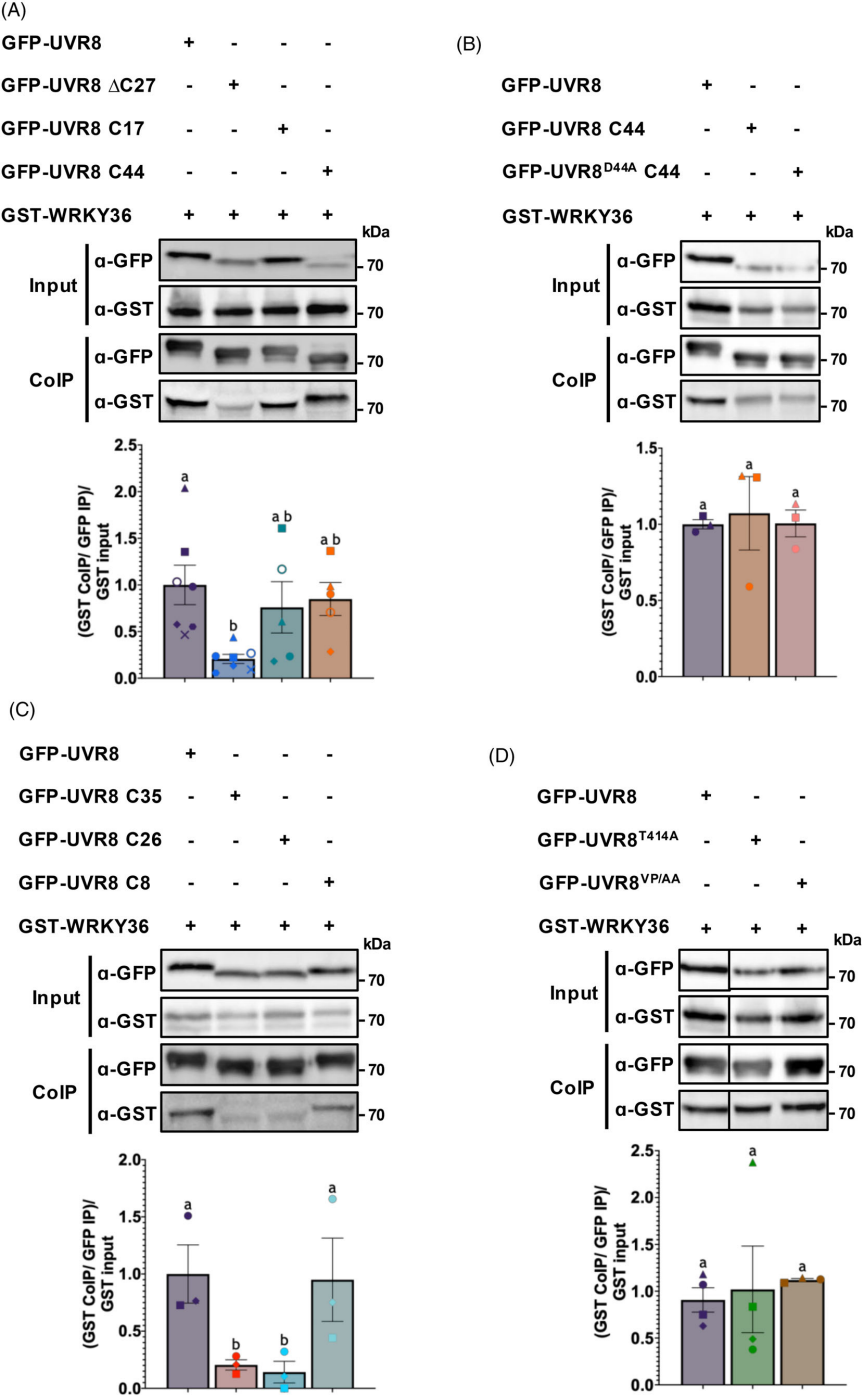
WRKY36 interacts with UVR8 C‐terminus residues 415–423. GFP‐UVR8 (WT or mutants indicated in A–D) and GST‐WRKY36 were transiently expressed in *Nicotiana benthamiana* leaves. After 60 h plants were exposed to UV‐B for 3 h. Relative expression (Input) was assayed via Western blot using anti‐GFP (ɑ‐GFP) and anti‐GST (ɑ‐GST) antibodies. GFP‐UVR8 was immunoprecipitated from the extract and the amounts of immunoprecipitated GFP‐UVR8 (CoIP, ɑ‐GFP) and co‐immunoprecipitated GST‐WRKY36 (CoIP, ɑ‐GST) were assayed. The upper section of each panel shows representative Western blot images and the lower section shows quantification of multiple repeats of each experiment; the bars in each graph correspond to the lanes above. For quantification, the intensity of the GST‐WRKY36 CoIP band was divided by that of the corresponding GFP‐UVR8 IP band and GST‐WRKY36 Input band. Each data point was normalised to the mean intensity value for WT GFP‐UVR8, set to 1. In each graph, data points sharing the same symbol were obtained in the same experiment. The data were analysed using either a repeated measures one‐way ANOVA (B, C) or a mixed‐effects analysis (A, D), both with Tukey's multiple comparison tests. Data are shown ± SE. Data points significantly different from each other (*P* < 0.05) are indicated by different letters above the bars. (A) GFP‐UVR8, GFP‐UVR8∆C27 *n* = 7, GFP‐UVR8 C17, GFP‐UVR8 C44 *n* = 5; (**P < 0.01). (B) GFP‐UVR8, GFP‐UVR8 C44, GFP‐UVR8 D44A C44 *n* = 3. (C) GFP‐UVR8, GFP‐UVR8 C35, GFP‐UVR8 C26, GFP‐UVR8 C8 *n* = 3; (*P < 0.05). (D) GFP‐UVR8, GFP‐UVR8 T414A *n* = 4, GFP‐UVR8 VPAA *n* = 3.

Interaction with WRKY36 was not significantly affected by the C17 deletion compared with wild‐type UVR8, whereas the ΔC27 deletion showed much reduced binding (Figure 2A). Surprisingly, the C44 deletion showed an interaction in multiple experiments (Figure 2A). A possible explanation is that WRKY36 binds preferentially to the C27 region but, when the entire C‐terminal region is removed, the protein is able to bind to a surface in the β‐propellor core domain of UVR8. Yang et al. (2018) also observed some interaction with the core domain in *in vitro* pull‐down assays.

Alphafold 3 (Abramson et al., 2024) was used to predict potential sites of interaction between UVR8 and WRKY36. However, since the C‐terminal domain of UVR8 is disordered and no crystal structure is available (Camacho et al., 2019; Christie et al., 2012; Wu et al., 2012), Alphafold was unable to confidently predict a structure for the C‐terminus and to model binding in this region. Nevertheless, it was possible to model interaction between WRKY36 and the β‐propellor core domain. We focused on the region of the Alphafold predicted alignment error (PAE) plot with the lowest score (Figure S1) and identified UVR8 amino acids that could potentially form H bonds with WRKY36 (Figure S1). The most confident region of the Alphafold models is where WRKY36 is close to the UVR8 dimer interface. In this region, UVR8 D44 was the only amino acid predicted to form hydrogen bonds with WRKY36 in all five Alphafold models. We therefore tested this possibility with the D44A mutation in the context of the C44 deletion mutant where binding to the core domain was observed, but the result showed that the mutation did not affect binding of WRKY36 to UVR8 C44 (Figure 2B).

Additional deletions within the C‐terminal region, lacking amino acids 406–440 and 415–440 (C35 and C26 respectively; Figure 1), were tested to try to further define the binding site within the C‐terminus. Neither mutant showed significant binding in *Nicotiana* CoIP assays (Figure 2C). Since interaction was seen with C17, lacking amino acids 424–440, the results suggest that WRKY36 may bind to UVR8 in the 415–423 region. Consistent with this interpretation and the result for C17, interaction was seen with the C8 mutant (lacking amino acids 433–440; Figure 2C).

Further mutants were assayed to test the above model. As predicted, a mutant of T414A was unaltered in the interaction (Figure 2D). In addition, we tested whether the VP motif, which is critical for binding COP1 and RUP proteins (Lau et al., 2019; Wang et al., 2022; Wang et al., 2023; Yin et al., 2015), is involved in the interaction; notably, the UVR8^V410A,P411A^ (VP/AA) mutant clearly retained interaction with WRKY36 (Figure 2D). We also previously reported that UVR8 can be phosphorylated within the C‐terminal region, but that phosphorylation of the main site, S402, had no effect on interaction with WRKY36 (Liu et al., 2024).

Finally, we decided to examine whether the results obtained in *Nicotiana* CoIP assays could be reproduced in a non‐plant system, namely, mammalian cells. This system gave similar results to *Nicotiana* when we tested S402 mutants (Liu et al., 2024) and, as a further control to validate the system, we observed that the ΔC27 mutant has much reduced interaction with RUP2, as expected from previous research (Cloix et al., 2012; Wang et al., 2023; Yin et al., 2015) (Figure S2). However, data for interaction of the C‐terminal mutants with WRKY36 were not entirely consistent with the *Nicotiana* results. An interaction was observed with the C17 and C44 mutants, consistent with *Nicotiana*, but an interaction was unexpectedly observed with the C26 (415–440) mutant (Figure S3). This observation could possibly be explained by differences in the conformation of the disordered C‐terminal region presented to WRKY36 in the animal and plant cell environments, but it highlights the potential problems of using non‐plant systems to draw conclusions about interactions in plant cells, where the presence of other molecules may influence protein conformation and protein interactions.

### BIM1 interacts with the 424–435 region of the UVR8 C‐terminus

**Figure 3:**
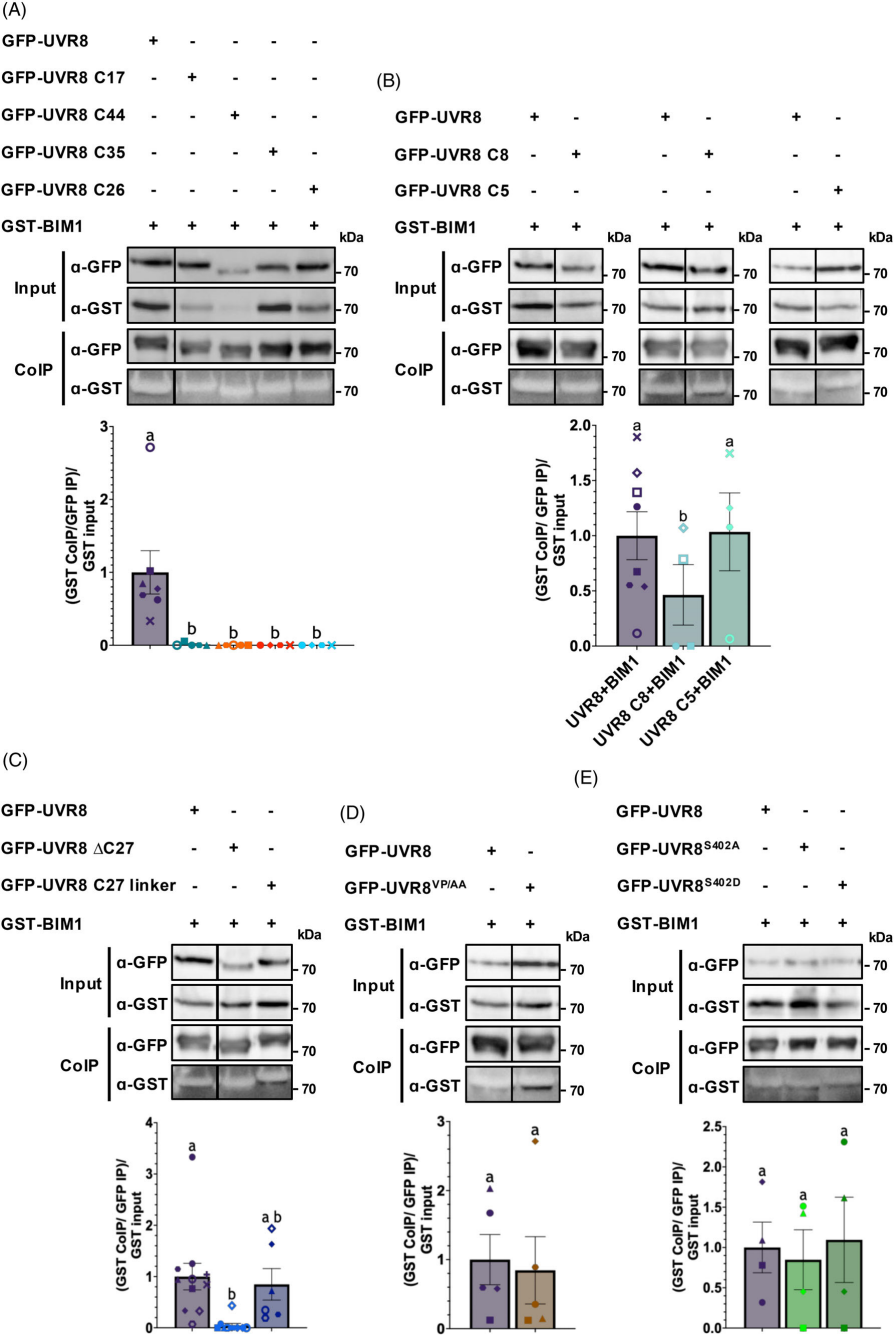
BIM1 interacts with UVR8 C‐terminus residues 424–435. GFP‐UVR8 (WT or mutants indicated in A–E) and GST‐BIM1 were transiently expressed in *Nicotiana benthamiana* leaves. After 60 hplants were exposed to UV‐B for 3 h. Relative expression (Input) was assayed via Western blot using anti‐GFP (ɑ‐GFP) and anti‐GST (ɑ‐GST) antibodies. GFP‐UVR8 was immunoprecipitated from the extract and the amounts of immunoprecipitated GFP‐UVR8 (CoIP, ɑ‐GFP) and co‐immunoprecipitated GST‐BIM1 (CoIP, ɑ‐GST) were assayed. The upper section of each panel shows representative Western blot images and the lower section shows quantification of multiple repeats of each experiment; the bars in each graph correspond to the lanes above. For quantification, the intensity of the GST‐BIM1 CoIP band was divided by that of the corresponding GFP‐UVR8 IP band and GST‐BIM1 Input band. Each data point was normalised to the mean intensity value for WT GFP‐UVR8, set to 1. In each graph data points sharing the same symbol were obtained in the same experiment. The data were analysed using either a repeated measures one‐way ANOVA (E), a mixed‐effects analysis (A, B, C), both with Tukey's multiple comparison tests, or a paired *t*‐test (D). Data are shown ± SE. Data points significantly different from each other (*P* < 0.05) are indicated by different letters above the bars. (A) GFP‐UVR8 *n* = 7, GFP‐UVR8 C17, GFP‐UVR8 C44 *n* = 5, GFP‐UVR8 C35, GFP‐UVR8 C26 *n* = 4; (**P < 0.01). (B) GFP‐UVR8 *n* = 8, GFP‐UVR8 C8, GFP‐UVR8 C5 *n* = 4; (*P < 0.05). (C) GFP‐UVR8, GFP‐UVR8 ∆C27 *n* = 11, GFP‐UVR8 C27 linker *n* = 6; (**P < 0.01). (D) GFP‐UVR8, GFP‐UVR8 VPAA *n* = 5. (E) GFP‐UVR8, GFP‐UVR8 S402A, GFP‐UVR8 S402D *n* = 4.

Liang et al. (2018) reported that BIM1 interacts with the C‐terminal region of UVR8 in yeast. We examined the interaction of BIM1 with the UVR8 C‐terminal deletion mutants in *Nicotiana*. The deletions C44, C35, C26 and C17 all lacked interaction (Figure 3A), whereas the C8 deletion (lacking amino acids 433–440) showed interaction in 2 of 4 experiments, and the C5 deletion (lacking 436–440) retained interaction in several repetitions (Figure 3B). These results point to interaction within the C‐terminal region, between approximately amino acids 424 and 435. However, the ΔC27 deletion lacked interaction (Figure 3C) even though it retains amino acids 424–440. A possible explanation is that interaction is disrupted because the internal deletion moves the binding site closer to the β‐propellor core domain, thereby modifying the steric interactions between the proteins. To test this possibility, we synthesised a 27 amino acid linker and used it to replace the 397–423 region in the C‐terminus. This linker was designed to have a similar balance of polar/non‐polar amino acids as C27 (see Methods). When we tested this variant, we found that it did interact with BIM1 (Figure 3C). Together the results suggest that BIM1 does not interact with the 397–423 region of UVR8, but it does interact with the 424–435 region of the C‐terminus. Consistent with this hypothesis, the VP/AA mutant retained interaction with BIM1 (Figure 3D). Likewise, there was no effect of the S402A phosphonull and S402D phosphomimetic mutations (Figure 3E).

We tested the interaction of selected UVR8 mutants with BIM1 in mammalian cells. Expression of BIM1 was weak, but some signal was observed with the C27 mutant and a clear interaction was seen with C8. Hence, the results are largely consistent with the *Nicotiana* data (Figure S4).

### The C‐terminal region of UVR8 is not required for interaction with BES1 and MYB13

**Figure 4:**
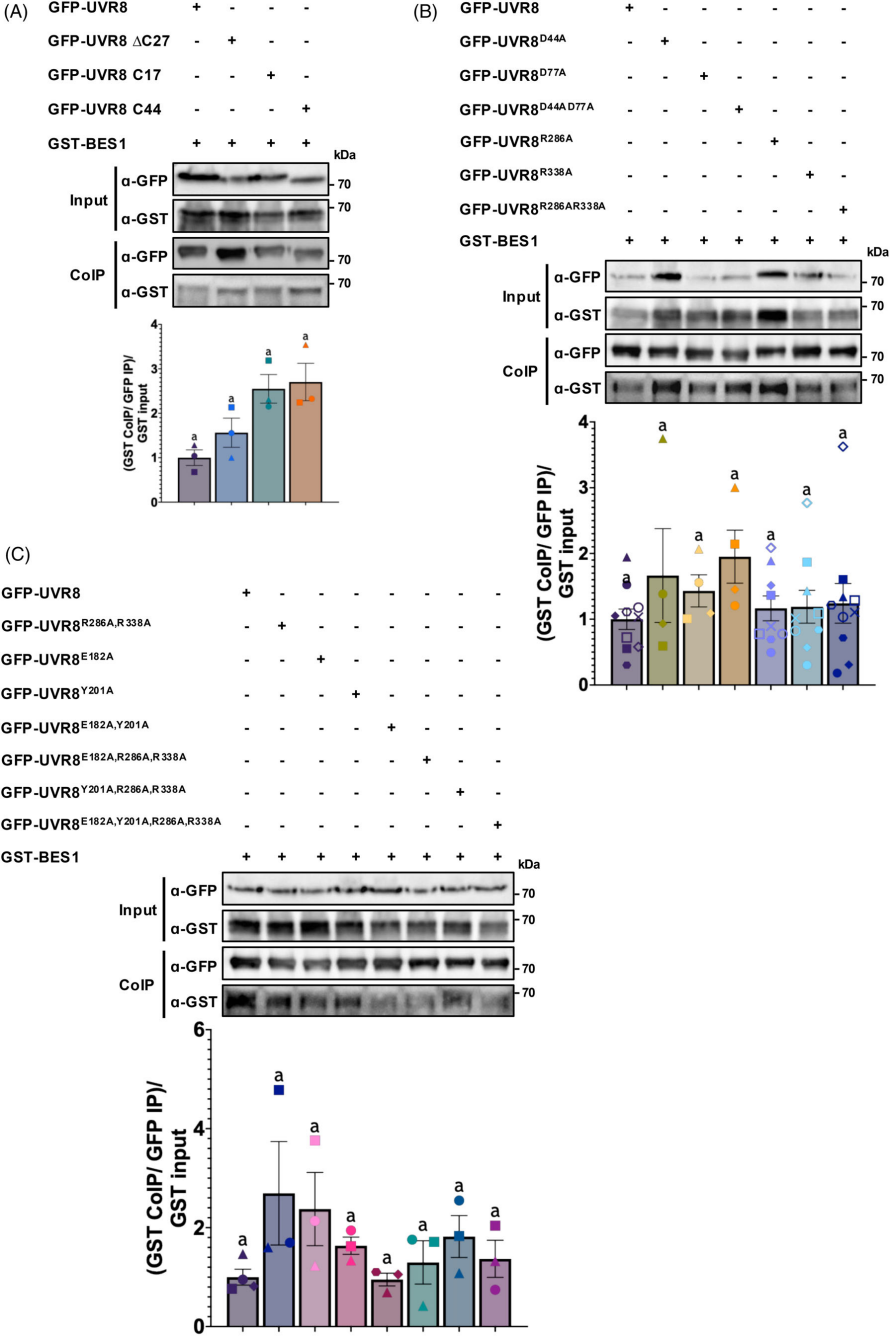
BES1 interaction with UVR8 does not require the C‐terminus. GFP‐UVR8 (WT or mutants indicated in A–C) and GST‐BES1 were transiently expressed in *Nicotiana benthamiana* leaves. After 60 h, plants were exposed to UV‐B for 3 h. Relative expression (Input) was assayed via Western blot using anti‐GFP (ɑ‐GFP) and anti‐GST (ɑ‐GST) antibodies. GFP‐UVR8 was immunoprecipitated from the extract and the amounts of immunoprecipitated GFP‐UVR8 (CoIP, ɑ‐GFP) and co‐immunoprecipitated GST‐BES1 (CoIP, ɑ‐GST) were assayed. The upper section of each panel shows representative Western blot images and the lower section shows quantification of multiple repeats of each experiment; the bars in each graph correspond to the lanes above. For quantification, the intensity of the GST‐BES1 CoIP band was divided by that of the corresponding GFP‐UVR8 IP band and GST‐BES1 Input band. Each data point was normalised to the mean intensity value for WT GFP‐UVR8, set to 1. In each graph data points sharing the same symbol were obtained in the same experiment. The data were analysed using either a repeated measures one‐way ANOVA (A) or a mixed‐effects analysis (B, C), both with Tukey's multiple comparison tests. Data are shown ± SE. Data points significantly different from each other (*P* < 0.05) are indicated by different letters above the bars. (A) GFP‐UVR8, GFP‐UVR8 ∆C27, GFP‐UVR8 C17, GFP‐UVR8 C44 *n* = 3; (*P < 0.05). (B) GFP‐UVR8, GFP‐UVR8 R286A R338A *n* = 10, GFP‐UVR8 D44A, GFP‐UVR8 D77A, GFP‐UVR8 D44A D77A *n* = 4, GFP‐UVR8 R286A, GFP‐UVR8 R338A *n* = 9. (C) GFP‐UVR8 *n* = 4, GFP‐UVR8 R286A R338A, GFP‐UVR8 E182A, GFP‐UVR8 Y201A, GFP‐UVR8 E182A Y201A, GFP‐UVR8 E182A R286A R338A, GFP‐UVR8 Y201A R286A R338A, GFP‐UVR8 E182A Y201A R286A R338A *n* = 3.

Liang et al. (2018) did not report whether BES1 interacted with any particular region of UVR8. In *Nicotiana*, we did not see any effect of the C‐terminal deletions on interaction with BES1 (Figure 4A). To some extent, binding was even stronger with the deletions than with the wild‐type fusion; the overall effect of deletions on binding was statistically significant and the *P* value obtained for the difference between wild‐type and C44 was 0.055. The results indicate that the C‐terminus is not required for interaction with BES1, suggesting that interaction may be with the β‐propellor core domain. Alphafold models suggest that interaction could be with the dimer interface, which is the region where interaction occurs with COP1 and RUP2 (Wang et al., 2022; Wang et al., 2023). We therefore tested several mutations of charged interface amino acids. We selected D44, D77, R286 and R338, as they are found in the interaction surfaces for both COP1 and RUP2 (Wang et al., 2022, Wang et al., 2023), while E182 and Y201 were suggested by Alphafold models (Figure S5). Alanine mutants of these amino acids were tested both singly and in combination. In some experiments, a small decrease in binding of BES1 was observed with the R286A and R338A single and double mutants (Figure 4B), but the E182A and Y201A single and double mutants, E182A, R286A, R338A, and Y201A, R286A, R338A triple mutants, and the E182A, Y201A, R286A, R338A quadruple mutant showed no obvious effects (Figure 4C) identified by this approach.

**Figure 5:**
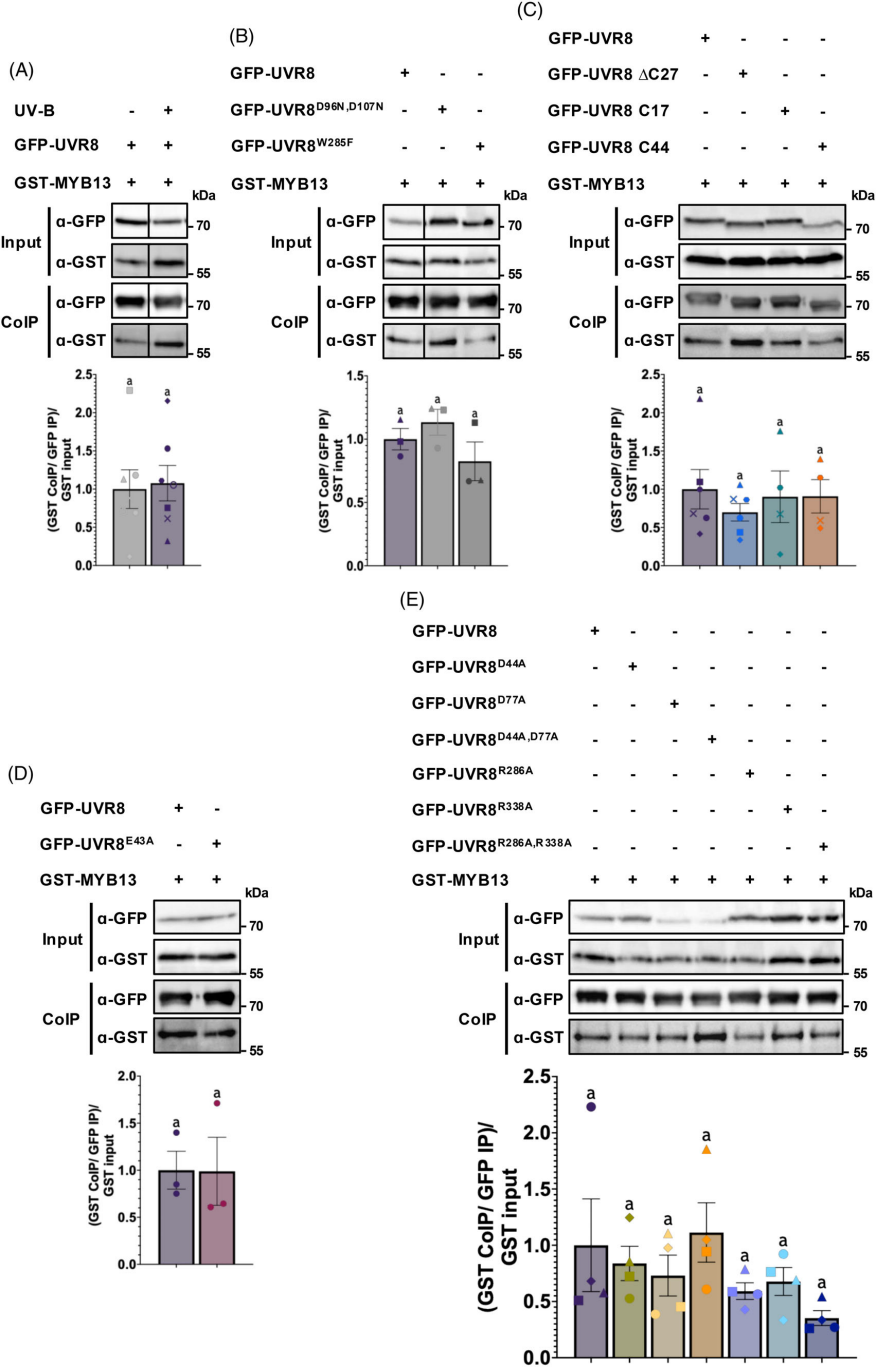
MYB13 interaction with UVR8 does not require the C‐terminus. GFP‐UVR8 (WT or mutants indicated in A–E) and GST‐MYB13 were transiently expressed in *Nicotiana benthamiana* leaves. After 60 h, plants were exposed to UV‐B for 3 h. Relative expression (Input) was assayed via Western blot using anti‐GFP (ɑ‐GFP) and anti‐GST (ɑ‐GST) antibodies. GFP‐UVR8 was immunoprecipitated from the extract and the amounts of immunoprecipitated GFP‐UVR8 (CoIP, ɑ‐GFP) and co‐immunoprecipitated GST‐MYB13 (CoIP, ɑ‐GST) were assayed. The upper section of each panel shows representative Western blot images and the lower section shows quantification of multiple repeats of each experiment; the bars in each graph correspond to the lanes above. For quantification, the intensity of the GST‐MYB13 CoIP band was divided by that of the corresponding GFP‐UVR8 IP band and GST‐MYB13 Input band. Each data point was normalised to the mean intensity value for WT GFP‐UVR8, set to 1. In each graph data points sharing the same symbol were obtained in the same experiment. The data were analysed using either a repeated measures one‐way ANOVA (C, E), a mixed‐effects analysis (B), both with Tukey's multiple comparison tests, or a paired *t*‐test (A, D). Data are shown ± SE. Data points significantly different from each other (*P* < 0.05) are indicated by different letters above the bars. (A) GFP‐UVR8 + UV‐B, GFP‐UVR8 − UV‐B *n* = 7. (B) GFP‐UVR8, GFP‐UVR8 D96N D107N, GFP‐UVR8 W285F *n* = 3. (C) GFP‐UVR8, GFP‐UVR8 ∆C27 *n* = 6, GFP‐UVR8 C17, GFP‐UVR8 C44 *n* = 4. (D) GFP‐UVR8, GFP‐UVR8 E43A *n* = 3. (E) GFP‐UVR8, GFP‐UVR8 D44A, GFP‐UVR8 D77A, GFP‐UVR8 D44A D77A, GFP‐UVR8 R286A, GFP‐UVR8 R338A, GFP‐UVR8 R286A R338A *n* = 4.

Qian et al. (2020) did not provide any information on the site of interaction of MYB13 with UVR8. They did report that interaction was stimulated by exposure to UV‐B, but we did not see a clear difference under our experimental conditions (Figure 5A). However, in each of our experiments there was greater binding of MYB13 to the constitutively monomeric D96N, D107N mutant than the constitutively dimeric W285F (Figure 5B), although the difference was not statistically significant. As with BES1, none of the C‐terminal deletions affected interaction between UVR8 and MYB13 (Figure 5C), indicating that the C‐terminus is not required for the interaction. Alphafold models were again used to suggest possible sites of interaction (Figure S6). We tested the possible involvement of E43, which was suggested by all of the models, but the E43A mutant did not affect the interaction (Figure 5D). Similarly, most of the other dimer interface mutants studied with BES1 were without effect, although there was some reduced interaction between MYB13 and R286A, R338A (Figure 5E). Therefore, although we can conclude that the C‐terminus is not required, we are unable to define in detail how MYB13 interacts with UVR8.

### Ratiometric bimolecular fluorescence complementation (rBiFC) data are consistent with co‐immunoprecipitation data for UVR8‐WRKY36 and UVR8‐BIM1 interaction sites

**Figure 6:**
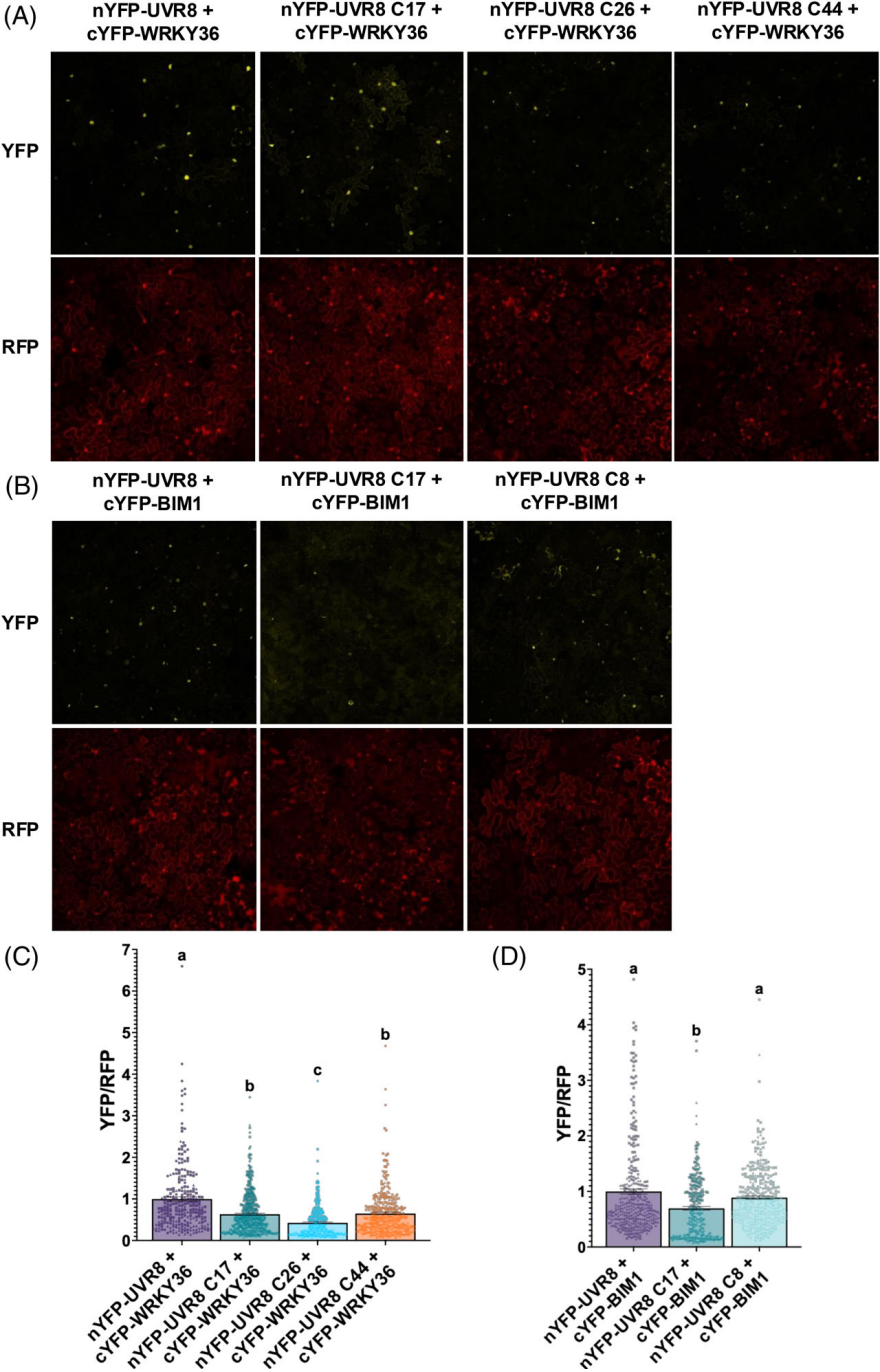
rBiFC data support the proposed UVR8‐WRKY36 and UVR8‐BIM1 interaction sites. nYFP‐UVR8 (WT or mutants indicated in A–D) and cYFP‐WRKY36 or cYFP‐BIM1 (as indicated in A–D) were transiently expressed in *Nicotiana benthamiana* leaves together with full‐length RFP as a transformation control. After 60 h plants were exposed to UV‐B for 3 h. Two leaf discs were excised from each transformed leaf and imaged using a confocal microscope for YFP and RFP fluorescence. Two images were taken per pair of leaf discs from randomly selected areas. (A–B) Representative images of the YFP and RFP fluorescence from leaves expressing each combination of UVR8 (WT or mutant) and either TF. (C–D) YFP and RFP fluorescence from the nuclei within the imaged area was quantified. Each data point represents an individual nucleus and nuclei from each biological replicate are shown with different symbol shapes. Each data point was normalised to the mean intensity value for the corresponding nYFP‐UVR8 + cYFP‐TF pair, set to 1. The data was analysed using an ordinary one‐way ANOVA with Tukey's multiple comparison test. Data are shown ± SE. Data sets significantly different from each other (*P* < 0.05) are indicated by different letters above the bars. (C) nYFP‐UVR8 + cYFP‐WRKY36 *n* = 251, nYFP‐UVR8 C17 + cYFP‐WRKY36 *n* = 452, nYFP‐UVR8 C26 + cYFP‐WRKY36 *n* = 334, nYFP‐UVR8 C44 + cYFP‐WRKY36 *n* = 337. (D) nYFP‐UVR8 + cYFP‐BIM1 *n* = 339, nYFP‐UVR8 C17 + cYFP‐BIM1 *n* = 282, nYFP‐UVR8 C8 + cYFP‐BIM1 *n* = 339. All significant differences showed *P* < 0.0001 with the exception of nYFP‐UVR8 C17 + cYFP‐BIM1 versus nYFP‐UVR8 C8 + cYFP‐BIM1 where *P* = 0.0010.

We used an additional plant‐based method, distinct from co‐immunoprecipitation, to test our conclusions regarding transcription factor interaction sites with UVR8. A ratiometric BiFC assay (Grefen & Blatt, 2012) was used to monitor interaction between key mutants of UVR8 and the WRKY36 and BIM1 transcription factors. A single vector carrying cassettes expressing fusions of wild‐type and mutant UVR8 with nYFP, fusions of the transcription factors with cYFP, and full‐length RFP as a transformation control was transfected into *Nicotiana* leaves. All the nuclei within the analysed tissue area were identified in the RFP fluorescence image. The levels of YFP fluorescence resulting from UVR8‐transcription factor interaction were then quantified in hundreds of identified nuclei and normalised to the fluorescence of co‐expressed RFP (Figure 6). For WRKY36, similar levels of YFP/RFP fluorescence were observed with the C17 and C44 deletions, which gave slightly lower fluorescence than wild‐type, and reduced fluorescence was observed with C26, consistent with the data in Figure 2. The slight but statistically significant difference observed between C17/C44 and WT UVR8 using rBiFC could be explained by the much greater number of data points afforded by this technique. This might allow small differences in interaction strength between the C‐terminus and core domain in the case of C44, or small effects due to conformational variation between WT and the C17 mutant to be more easily detected. For BIM1, C17 showed weaker fluorescence than wild‐type, consistent with the data in Figure 3. Fluorescence was observed with the C8 mutant, which showed variable interaction in *Nicotiana* co‐immunoprecipitation assays (Figure 3) and mammalian cells (Figure S4). A likely explanation of the inconsistent results with the C8 mutant is that the end point of the C8 mutant sequence (amino acid 433) is at the distal end of the BIM1 interaction site. Overall, the rBiFC data support the findings from the co‐immunoprecipitation assays in *Nicotiana*.

## DISCUSSION

The data presented in this study provide new insights into how transcription factors interact with UVR8. These interactions are pivotal in determining the nature of responses mediated by the photoreceptor. Previous research in Arabidopsis has highlighted the physiological importance of interactions of UVR8 with various transcription factors, including those studied here. WRKY36 represses transcription of the *HY5* gene, and hence its interaction with UVR8 following UV‐B exposure promotes HY5‐mediated transcription of many UVR8‐target genes and the suppression of hypocotyl extension in UV‐B (Yang et al., 2018). Similarly, the binding of BES1 and BIM1 to UVR8 following UV‐B exposure contributes to the suppression of extension growth by modifying transcription of gene targets of brassinosteroid signalling (Liang et al., 2018). In addition, interaction of UVR8 with MYB13 modifies transcription of genes involved in flavonoid biosynthesis, lateral root growth and cotyledon expansion (Qian et al., 2020). However, although the above interactions have major physiological consequences, very little information was reported on the sites of interaction of the transcription factors with UVR8. Identifying these sites is therefore a critical first step in bridging the gap between the molecular interaction and physiological outcomes, which could then allow the tuning of these responses in crop species.

Our aim in this study was to examine UVR8‐transcription factor interactions using plant material because heterologous and *in vitro* systems do not always reflect the *in planta* situation. We chose to use the co‐immunoprecipitation assay, as in our experience, it can produce quantitative data (Liao et al., 2020; Liu et al., 2024). Nevertheless, the assay is inherently variable because it depends on expression of two separate fusions, which may occur to different extents, and there are differences in the efficiency of transient expression between *Nicotiana* leaves of the same plant and between different plants. In addition, the binding efficiencies, elution and immuno‐detection processes can also introduce variability. Under the conditions used, we observed that the level of GFP‐UVR8 expression was not correlated with the GST‐transcription factor CoIP signal unless expression was very weak, suggesting that the methods employed provided a consistent level of bait protein. Therefore, the CoIP signal was quantified relative to the level of immunoprecipitated GFP‐UVR8 rather than the GFP‐UVR8 ‘Input’ level. Similarly, expression of the GST‐transcription factor fusion only affected binding to immobilised GFP‐UVR8 if it was below a threshold, but some effect was observed quite frequently. To determine the best way to quantify the data, we calculated GST‐transcription factor CoIP/GFP‐UVR8 IP, either normalised or not normalised, to the input of the GST‐transcription factor fusion, but there was no clear difference in the results obtained with these methods. Nevertheless, to counter any possible effect of variation in GST‐transcription factor expression, all the data presented in the figures are normalised to the GST‐transcription factor ‘input’ level.

To examine the binding sites for transcription factors, our initial strategy was to assay interaction with C‐terminal deletion mutants of UVR8 lacking regions likely to be important: the C‐terminus as a whole, the C27 region and C17. Further deletions allowed us to examine binding to smaller sections of the C‐terminus, and various point mutations of specific amino acids were also assayed, guided either by knowledge of COP1/RUP interactions or by Alphafold models. Alphafold was able to suggest possible regions of interaction with the UVR8 core domain, but was unable to predict binding sites in the disordered C‐terminal region.

The data presented here indicate that WRKY36 binds to the C‐terminal region of UVR8, in agreement with the observations of Yang et al. (2018). Our results with deletion mutants extend the previous report as they suggest that a region between amino acids 415–423 is required for the WRKY36 interaction. Consistent with this proposal, mutations of T414 and the VP motif (V410 and P411) did not affect the interaction. Whereas the deletion experiments indicate that WRKY36 interacts with the C‐terminal region, there is evidence for some interaction with the UVR8 β‐propellor core domain. We observed binding when the entire C‐terminal region was deleted (the C44 mutant), consistent with the previous observation of residual binding (Yang et al., 2018). However, further experiments are required to determine the strength and site of interaction with the core domain. Alphafold models raised the possibility of WRKY36 binding to the dimer interaction surface, but mutation of D44, which was suggested to form hydrogen bonds with WRKY36, had no effect. More detailed mutant analysis will be required to investigate this potential binding site.

Our study indicates that BIM1 interacts with the C‐terminus of UVR8, consistent with the yeast two‐hybrid data of Liang et al. (2018). In mutants which lacked binding (Figure 4A) there was no obvious residual interaction, suggesting very little if any binding to the core domain. Deletion analysis indicated that BIM1 binds in the 424–435 amino acid region of UVR8: the C17 mutant (lacking amino acids 424–440) showed no interaction, whereas C5 (lacking amino acids 436–440) retained binding; C8 (lacking amino acids 433–440) lacked binding in some but not all experiments. As with WRKY36, mutation of the VP motif had no effect on binding.

The results obtained in co‐immunoprecipitation experiments for binding of WRKY36 and BIM1 to UVR8 are supported by the rBiFC data. We chose to use the rBiFC assay because it tests interaction in plants and is not based on co‐immunoprecipitation. Moreover, in comparison to standard BiFC assays, rBiFC includes the addition of an internal RFP transformation control to account for variation in transfection efficiency. This allows more reliable quantification if sufficient nuclei are quantified and YFP fluorescence is normalised against that of RFP. In addition, a large number of nuclei can be identified in each image, which allows for large numbers of individual data points to be obtained thus yielding the potential to statistically distinguish smaller differences in interaction strength. Nevertheless, the large number of data points does not correspond to the same number of true biological replicates. Therefore, it is important to confirm any statistically significant but slight differences with alternative techniques such as co‐immunoprecipitation. In addition, although one can be sure that the *Agrobacterium* did transform all three fusion cassettes in any cell expressing RFP, this does not necessarily guarantee that the expression levels of all three will be equal as, although they all use the same promoter, post‐transcriptional and post‐translational effects on each fusion might cause differences in protein level, whereas any differences in protein expression can be detected and normalised in co‐immunoprecipitation assays. Lastly, imaging is undertaken for small, randomly selected areas of the leaf, and it is difficult to know if these are truly representative of the whole leaf. This consideration is avoided when analysing expression and interaction in the entire leaf through co‐immunoprecipitation assays.

In contrast to WRKY36 and BIM1, there is no evidence from deletion analysis that BES1 and MYB13 interact with the UVR8 C‐terminus. In fact, there is some suggestion that the C‐terminus impairs the interaction with BES1. Previous studies (Liang et al., 2018; Qian et al., 2020) do not provide any information on the sites of interaction. Based on the knowledge of COP1 and RUP binding sites, the dimer interface is an attractive potential site of interaction, and the Alphafold models support this possibility. However, experiments with point mutations in several candidate amino acids did not provide evidence in support of the model, although a small decrease in binding was observed in some assays of BES1 and MYB13 with mutants in R286 and R338. For all interactions with the core domain, it is likely that binding occurs to one or more patches of amino acids that provide the required chemical characteristics, and mutation of one or two of these residues may not be sufficient to cause substantial loss of interaction. Identification of key residues in UVR8 dimer formation, and in COP1 and RUP binding to the UVR8 dimer interaction surface, was informed by structural studies (Christie et al., 2012; Wang et al., 2022; Wang et al., 2023; Wu et al., 2012), which are lacking for the interaction with BES1 and MYB13.

**Figure 7:**
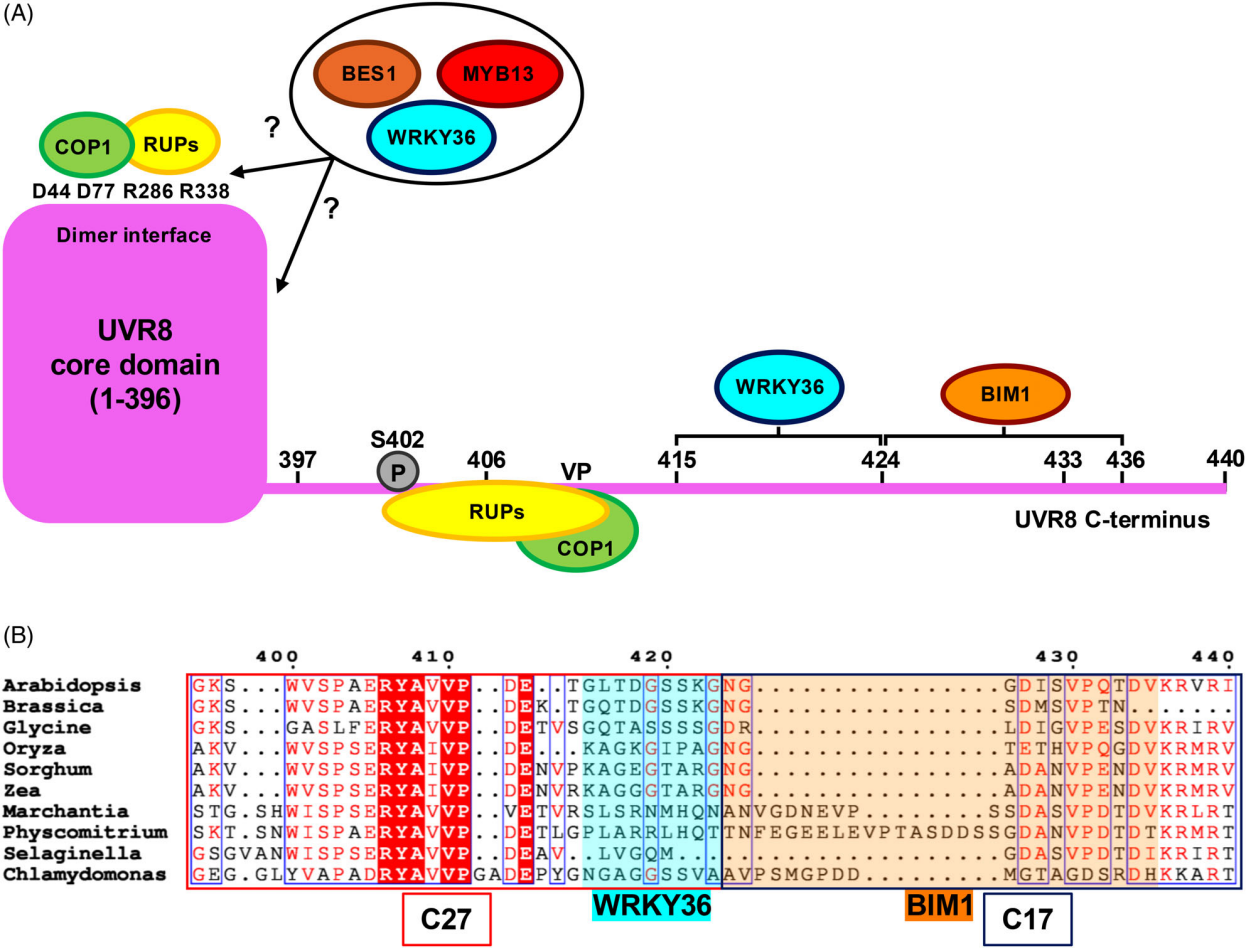
Model of UVR8 interactions with transcription factors. (A) Diagram showing the regions identified in this study where WRKY36 and BIM1 interact with the UVR8 C‐terminus relative to interaction sites for RUPs and COP1 and relevant features of the region (S402 phosphorylation site and VP motif). C44 is divided into sections corresponding to the deletion mutants used. Interactions of COP1 and RUPs with the dimer interface and the hypothesised interactions of BES1 and MYB13 with the dimer interface and/or core domain are also shown. (B) Multiple sequence alignment of UVR8 C44 sequences from *Arabidopsis*, dicots *Brassica oleracea* and *Glycine max*, monocots *Oryza sativa* (subspecies *japonica*), *Sorghum bicolor* and *Zea mays*, bryophytes *Marchantia polymorpha* and *Physcomitrium patens*, lycophyte *Selaginella moellendorffii* and the single‐cell green alga *Chlamydomonas reinhardtii*. Completely conserved amino acids are highlighted in red, highly conserved ones are written in red and conserved regions are boxed in blue. The C27 and C17 regions are boxed in red and dark blue, respectively. The WRKY36 and BIM1 interaction sites are shaded in cyan and orange, respectively. Sequences are listed in order of most to least conserved with respect to *Arabidopsis*. The alignment was produced using M_ULT_A_LIN_ and the figure prepared using ESP_RIPT_ 3.0.

The present study shows that transcription factors interact with UVR8 in different ways (Figure 7), either specifically with the C‐terminus (BIM1), only with the core domain (MYB13 and BES1), or with both regions of the protein (WRKY36). Moreover, the deletion analysis indicates that WRKY36 and BIM1 require different regions of the C‐terminus for binding, as illustrated in Figure 7. In addition, it is particularly interesting that the binding sites for transcription factors in the C‐terminal region are distinct from those for COP1 and RUP proteins, which are well defined through structural studies (Wang et al., 2022, Wang et al., 2023). COP1 and RUPs have overlapping binding sites in the 400–413 region of C27, whereas the binding of WRKY36 and BIM1 is further towards the C‐terminus. Consistent with this model, mutational analysis shows that the binding of WRKY36 and BIM1 does not require the VP motif, which is critical for binding COP1 and RUPs. It remains to be determined whether any of the other transcription factors that interact with UVR8 bind to the same C‐terminal region as COP1 and RUP proteins.

The region of the C‐terminus where COP1 and RUP proteins bind is highly conserved between species, whereas the region where WRKY36 is proposed to bind is not; interestingly, the proposed site for BIM1 binding is quite well conserved. COP1 and RUP proteins function in UVR8 signalling in diverse plant species, whereas numerous transcription factors are likely to be involved in UVR8‐mediated responses. A number of transcription factors have been shown to bind to UVR8 and the number is likely to increase. We speculate that the region of the C‐terminus between amino acids 414 and 440 may provide interaction sites for several transcription factors in a given species, and that the low sequence conservation of this region between species may reflect the diversity in transcription factors mediating various responses to UV‐B. Transcription factor binding studies with UVR8 from a variety of species should provide insights into the functional significance of the C‐terminal sequences during evolution.

Although this study extends information on the interaction of transcription factors with UVR8, there is much that we do not know. Binding sites in the core domain have not been identified and it will be interesting to see whether these differ between transcription factors and whether they differ from those used by COP1 and RUP proteins. It may be that several proteins interact at the dimer interface. However, this surface will be hidden in the dimeric form, and this may explain why transcription factors such as MYB13 bind more strongly following UV‐B exposure (Qian et al., 2020). Interaction with BES1 is reported to be independent of UV‐B (Liang et al., 2018), suggesting that the binding site in the core domain may differ from that of MYB13. Furthermore, it is possible that proteins bind to both the dimer interface and some other part of the core domain, which presumably would affect the strength of binding to the dimeric and monomeric forms. Apart from the binding sites, several factors will influence whether UVR8 interacts with a specific transcription factor (Liu & Jenkins, 2024). First, the transcription factor may be subject to differential expression, which will determine whether it is present in particular cells or tissues. Second, binding will be dependent on the relative amounts and binding affinities of the various transcription factors present, but such information is lacking for proteins that interact with UVR8. Research to understand the basis of these interactions is vital given their crucial importance in directing UVR8‐mediated responses to UV‐B.

## EXPERIMENTAL PROCEDURES

### Plasmid construct design and cloning

Plasmids used here for expressing GFP‐UVR8 WT fusions in plants and mammalian cells based on the pEZR(K)L‐C or pcDNA3 vector, respectively, were previously described (Liu et al., 2024). Plasmids used for rBiFC carrying cassettes for expression of nYFP‐UVR8 and cYFP‐WRKY36 or cYFP‐BIM1 fusions, as well as RFP as a transformation control, were previously described in Grefen and Blatt (2012). The deletion mutants designed for this work were cloned into the same vector backbones using either In‐Fusion HD cloning (Takara Bio, San Jose, CA, USA) for pEZR(K)L‐C and pcDNA3 based vectors, or Gateway cloning (Invitrogen, Waltham, MA, USA) for rBiFC vectors following the manufacturers' instructions. Single/double‐residue mutagenesis was performed via PCR amplification of pSP64‐GFP‐UVR8 vectors using primers designed using either the Agilent QuikChange Primer Design or the in‐Fusion cloning primer design online tools. PCR amplification was performed using either Q5 High‐fidelity DNA polymerase (New England Biolabs, Ipswich, MA, USA, M0491) or KOD Hot‐start DNA polymerase (Sigma‐Aldrich, St. Louis, MO, USA, 71 086) following the manufacturers' instructions. The mutated fusions were then cloned into the plant or mammalian cells expression vectors as described above.

The GFP‐UVR8 C27 linker plasmid was designed to substitute the C27 region with a linker to maintain the distance between the C17 region and the core domain. The linker sequence used was the following: GTAGTAAGTGGVAGTAAGTGGTAGVAA. The sequence was designed based on a smaller, 12 residue linker used for GFP‐tagged proteins by Waldo et al. (1999): GSAGSAAGSGEF. Several modifications were, however, made to fit our purposes. First, the terminal EF residues were removed to avoid potential effects of large or charged side chains. The linker was then expanded to 27 residues to match the length of C27. Serine residues used in the linker to maintain some hydrophilicity to the region were substituted by threonines as serine phosphorylation plays an important role in UVR8 C27 interactions. Lastly, two of these threonines were substituted for valines to maintain the same number of polar residues as present in the C27. In the DNA sequence of the linker, the two most used codons in plants for each residue were alternated to avoid long sequence repetitions which would impair the synthesis of the linker.

### *Nicotiana benthamiana* transient transformation

Purified plasmid DNA was used to transform electrocompetent *Agrobacterium tumefaciens* cells via electroporation. The *N. benthamiana* plants used for transient transformation were grown for 3–5 weeks in long days (16 h light, 8 h darkness) under 50–100 μmol m^−2^ s^−1^ white light. *Agrobacterium* cells were resuspended at a calculated OD_600_ of 0.4 in infiltration buffer (10 mm MgCl_2_, 10 mm MES, pH 5.6, 200 μm acetosyringone). The solution was infiltrated into the abaxial side of the *Nicotiana* leaves using a needleless syringe. After the infiltration, the plants were returned to the growth chamber for approximately 60 h. Where indicated, the plants were exposed to 3 h of 3 μmol m^−2^ s^−1^ supplemental UV‐B light at the end of the incubation prior to protein extraction.

### Mammalian cell transfection

Human embryonic kidney (HEK) 293 T cells were cultured in Dulbecco's modified Eagle's medium (DMEM) (ThermoFisher, Waltham, MA, USA, 41 965 039) supplemented with 10% heat inactivated fetal bovine serum (FBS) (ThermoFisher, 10 500 064) and 100 units/ml penicillin/100 μg/ml streptomycin (Sigma‐Aldrich, P0781) in a humidified 5% (v/v) CO_2_ atmosphere at 37°C. The cells were then transfected with purified plasmid DNA via polyethylenimine (PEI, Polysciences, Warrington, PA, USA, 23 966) transfection as described previously (Liu et al., 2024). For each transfection, approximately 2 × 10^6^ cells were used. Following transfection, the cells were returned to the incubator for 48 h. They were then transferred to Hank's balanced salt solution (HBSS) (Gibco, Waltham, MA, USA, 14 065 072) supplemented with 0.35% sodium bicarbonate (Gibco, 25 080 094) and 10 mm HEPES, pH 7.4 to allow for them to be treated with UV‐B light outside of the incubator. The light treatment consisted of 4 h of 0.1 μmol m^−2^ s^−1^ UV‐B provided by narrowband UV‐B tubes (Philips TL20W/01RS, Aachen, Germany).

### Protein methods

Total protein extraction was performed as described previously (Liu et al., 2024). *N. benthamiana* leaf tissue was frozen and ground in liquid nitrogen. Total protein was extracted from the tissue powder using extraction buffer (25 mm Tris–HCl pH 7.5, 1 mm EDTA, 150 mm NaCl, 10% (v/v) glycerol, 0.1% (w/v) Nonidet‐P40, 10 mm DTT, cOmplete mini EDTA‐free protease inhibitor cocktail (Roche, Basel, Switzerland, 11 836 170 001), 0.5 mm PMSF). Mammalian cells were isolated from the media via centrifugation and total protein was extracted using lysis buffer (150 mm NaCl, 1% Triton X‐100, 50 mm Tris–HCl pH 8.0, cOmplete mini EDTA‐free protease inhibitor cocktail (Roche, 11 836 170 001), 0.5 mm PMSF). Co‐immunoprecipitation assays were performed as previously reported using GFP‐trap agarose beads (Chromotek, Rosemont, IL, USA, gta) (Liu et al., 2024). Input and co‐immunoprecipitated samples were then run on SDS‐PAGE gels and immunoblotted using standard methods (Liu et al., 2024). Images of the membranes were taken using a Fusion FX Spectra imager (Vilber, Paris, France). The antibodies used were: anti‐GFP (Proteintech, Rosemont, IL, USA, 3 h9), anti‐GST (Genscript, Nanjing, China, A00865), anti‐HA (Roche, 3F10), anti‐rat (Newmarket Scientific, Kentford, UK, AS10 1187), anti‐mouse (Promega, Madison, WI, USA, W4021).

### Western blot image quantification and statistical analysis

Quantification of bands in the Western blot images was performed using ImageJ software. The quantification was carried out by measuring the intensity of the signal for bands of the co‐immunoprecipitated GST‐TF and dividing it first by the signal intensity for the corresponding immunoprecipitated GFP‐UVR8 band and then by that of the corresponding GST‐TF input band. Each individual data point was then normalised to the mean intensity value for WT GFP‐UVR8, which is set to 1.

At least three biological replicates for each combination of GFP‐UVR8 WT/mutant and GST‐TF were performed and quantified. Where more than three data sets were compared, the data were analysed using either a repeated measures one‐way ANOVA or a mixed‐effects analysis, depending on whether each bar had the same number of biological repeats, both with Tukey's multiple comparison tests. If only two data sets were compared, a paired *t*‐test was used for the analysis. The analysis was performed using the GraphPad Prism 10 software.

### rBiFC assay and analysis

Two leaf discs were excised from each *N. benthamiana* leaf transformed with each rBiFC vector. The discs were imaged using a Leica TCS SP8 confocal laser scanning microscope (Leica, Wetzlar, Germany) with a HC PL APO 20×/0.75 lens. Fluorescence was excited at 514 nm for YFP and 552 nm for RFP. Fluorescence emission was collected between 521 and 553 nm for YFP and between 595 and 625 nm for RFP, using HyD SMD and PMT detectors, respectively. Two areas for each pair of leaf discs were randomly selected for imaging. Standardised settings were used for every sample. Z‐stacks of each region composed of 15 images taken at 2 μm intervals were obtained. Three biological replicates of the experiment were performed.

The Z‐stacks were processed as sum intensity projections using ImageJ software. The RFP image for each sample was used to define the regions of interest (ROIs) corresponding to all the nuclei within the tissue area. The integrated density of the fluorescence signal within the ROIs was measured in both the RFP and YFP images. The ratio between the YFP and RFP signals was then calculated and used as the final value to quantify the interaction within each nucleus. Data points for all nuclei within each pair of images for each biological replicate were pooled together and analysed using an ordinary one‐way ANOVA with Tukey's multiple comparison test. The analysis was performed using the GraphPad Prism 10 software.

### Alphafold analysis

Structures of interaction complexes between UVR8 and TFs were predicted using the Alphafold 3 model (Abramson et al., 2024). The sequences of the two proteins were supplied to the model and five different predicted structures were produced by the software. Together with the structures, predicted alignment error (PAE), predicted local distance difference test (pLDDT) and predicted Template Modelling (pTM) scores are also provided for each model. The PAE score is a measure of the confidence of each model in the position of any one residue with respect to every other residue in the structure. From these scores a plot can be produced that we used to identify patches of the TF predicted structures with the lowest scores and therefore highest confidence. In our case, these patches were conserved throughout the five models for each UVR8‐TF combination to varying degrees. We then used the UCSF ChimeraX (Meng et al., 2023) software to predict potential H bonds that could be formed between UVR8 and these higher confidence regions of the TFs. Residues that formed H bonds in all five models were selected for mutagenesis and *in vivo* interaction testing via CoIP assay.

### UVR8 sequence alignment

Sequences for the UVR8 proteins from various plant species were obtained from the UniProtKB database. A multiple sequence alignment was then produced with these using the M_ULT_A_LIN_ tool (Corpet, 1988). The ESP_RIPT_ 3.0 (Robert & Gouet, 2014) tool was then used to produce a figure of the aligned sequences for the C44 region of UVR8 across the various species.

## AUTHOR CONTRIBUTIONS

GIJ conceived the research. GG planned and designed the experiments with input from GIJ and WL. GG performed the experiments, analysed the data and produced the figures. Data were interpreted by GG together with GIJ and WL. GIJ and GG wrote the manuscript with input from WL.

## CONFLICT OF INTEREST

The authors declare no conflict of interest.

## Supporting information


**Figure S1.** Alphafold analysis of the UVR8‐WRKY36 interaction.
**Figure S2**. RUP2 interaction with UVR8 in mammalian cells requires the C27 region.
**Figure S3**. WRKY36 interaction with UVR8 C‐terminus in mammalian cells is not consistent with *in planta* observations.
**Figure S4**. BIM1 interaction with UVR8 in mammalian cells requires the C44 region.
**Figure S5**. Alphafold analysis of the UVR8‐BES1 interaction.
**Figure S6**. Alphafold analysis of the UVR8‐MYB13 interaction.

## Data Availability

The data that supports the findings of this study are available within the paper and the Supporting Information of this article.
