# Supplementary material for: Transcription factors interact with Arabidopsis UVR8 photoreceptor at distinct sites to COP1 and RUP proteins
Source: Plant J. 2026 Jul 1;127(1):e71009. doi: 10.1111/tpj.71009 (PMC13322755; doi:10.1111/tpj.71009)
Supplement: Supplementary file 1 — Figure S1. Alphafold analysis of the UVR8‐WRKY36 interaction. Figure S2. RUP2 interaction with UVR8 in mammalian cells requires the C27 region. Figure S3. WRKY36 interaction with UVR8 C‐terminus in mammalian cells is not consistent with in planta observations. Figure S4. BIM1 interaction with UVR8 in mammalian cells requires the C44 region. Figure S5. Alphafold analysis of the UVR8‐BES1 interaction. Figure S6. Alphafold analysis of the UVR8‐MYB13 interaction. [file TPJ-127-0-s001.pdf]

Supporting information

Transcription factors interact with Arabidopsis UVR8 photoreceptor at distinct sites to COP1 and RUP proteins

Giovanni Giuriani[1], Wei Liu[1], Gareth I. Jenkins[1]*

[1] School of Molecular Biosciences, College of Medical, Veterinary and Life Sciences, Bower Building, University of Glasgow, Glasgow G12 8QQ, UK

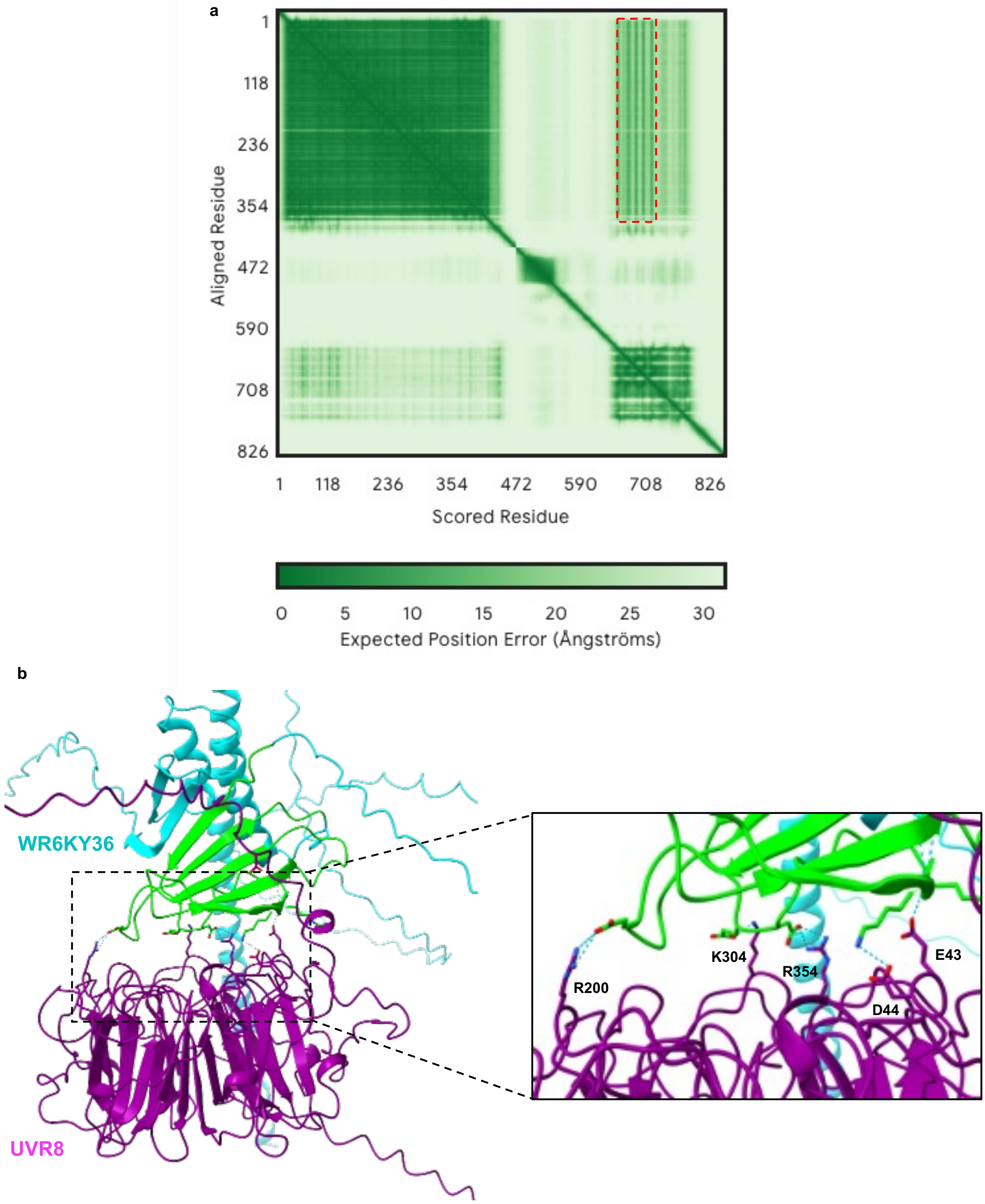

Figure S1 Alphafold analysis of the UVR8-WRKY36 interaction. The amino acid sequences of UVR8 and WRKY36 were fed to Alphafold 3 to obtain a predicted structure of the complex of the two proteins from which to derive potential interaction sites and interacting residues to be tested *in vivo.* The programme produced 5 models of the complex's structure. Here we show the results of the first of these models. (a) Predicted alignment error (PAE), also known as Expected Position Error, plot for the

model. This plots a score given by the program in Ångströms (Å) to each residue in both protein sequences relative to every other residue in both proteins that indicates how confident the model is of the predicted position of any one residue with respect to the rest of the structure. Lower scores indicate a more confident prediction. The red dashed box indicates the region of WRKY36 whose position was most confidently predicted relative to UVR8. We focused on this region to find potential residues within UVR8 that could be important in the interaction with WRKY36. (b) Structure prediction of the complex. On the left side of the panel is the predicted structure of the UVR8-WRKY36 complex in its entirety shown in cartoon form. The two proteins are shown in different colours as indicated. The green section indicates the region highlighted by the box in (a). On the right side of the panel is a zoomed-in view of the interface between the two proteins with the residues that could form potential H-bonds between the proteins labelled and shown in stick form (N atoms shown in blue and O atoms shown in red). The H-bonds are shown by blue dashed lines.

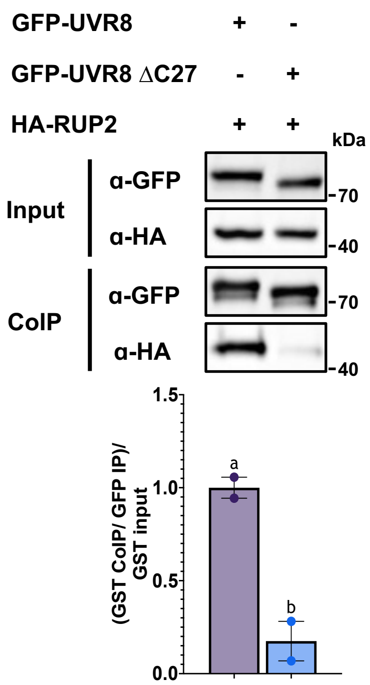

Figure S2 RUP2 interaction with UVR8 in mammalian cells requires the C27 region. GFP-UVR8 (WT or ΔC27 as indicated) and HA-RUP2 were transiently expressed in HEK 293T cells. After 48 hours the cells were exposed to UV-B for 4 hours. Relative expression (Input) was assayed via western blot using anti-GFP (α-GFP) and anti-HA (α-HA) antibodies. GFP-UVR8 was immunoprecipitated from the extract and the amounts of immunoprecipitated GFP-UVR8 (CoIP, α-GFP) and co-immunoprecipitated HA-RUP2 (CoIP, α-HA) were assayed. The upper section of each panel shows representative western blot images and the lower section shows quantification of multiple repeats of each experiment; the bars in each graph correspond to the lanes above. For quantification, the intensity of the HA-RUP2 CoIP band was divided by that of the corresponding GFP-UVR8 IP band and HA-RUP2 Input band. Each data point was normalised to the mean intensity value for WT GFP-UVR8, set to 1. In each graph data points sharing the same symbol were obtained in the same experiment. The data was analysed using a paired t-test. Data are shown ± S.E. Data points significantly different from each other ($p<0.05$) are indicated by different letters above the bars. GFP-UVR8, GFP-UVR8 ΔC27 n=2.

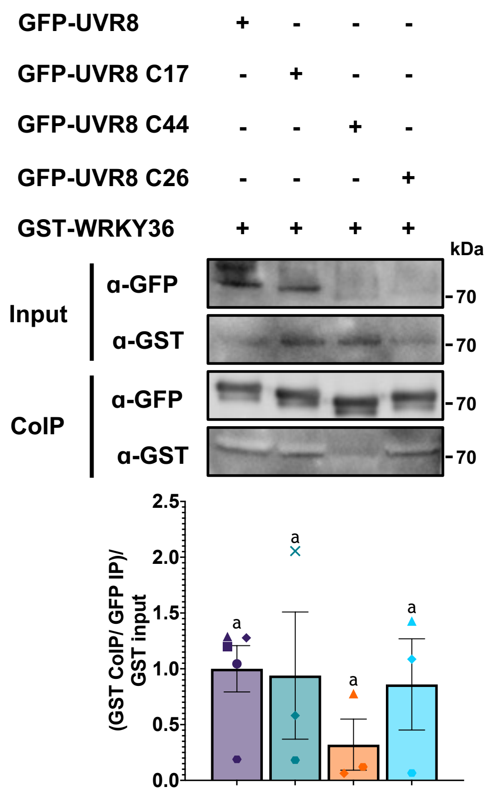

Figure S3 WRKY36 interaction with UVR8 C-terminus in mammalian cells is not consistent with *in planta* observations. GFP-UVR8 (WT or mutants as indicated) and GST-WRKY36 were transiently expressed in HEK 293T cells. After 48 hours the cells were exposed to UV-B for 4 hours. Relative expression (Input) was assayed via western blot using anti-GFP (α-GFP) and anti-GST (α-GST) antibodies. GFP-UVR8 was immunoprecipitated from the extract and the amounts of immunoprecipitated GFP-UVR8 (CoIP, α-GFP) and co-immunoprecipitated GST-WRKY36 (CoIP, α-GST) were assayed. The upper section of each panel shows representative western blot images and the lower section shows quantification of multiple repeats of each experiment; the bars in each graph correspond to the lanes above. For quantification, the intensity of the GST-WRKY36 CoIP band was divided by that of the corresponding GFP-UVR8 IP band and GST-WRKY36 Input band. Each data point was normalised to the mean intensity value for WT GFP-UVR8, set to 1. In each graph data points sharing the same symbol were obtained in the same experiment. The data was analysed using a mixed-effects analysis with Tukey's multiple comparison tests. Data are shown ± S.E. Data points significantly different from each other ($p<0.05$) are indicated by different letters above the bars. GFP-UVR8 n=5, GFP-UVR8 ΔC27, GFP-UVR8 C17, GFP-UVR8 C44 n=3.

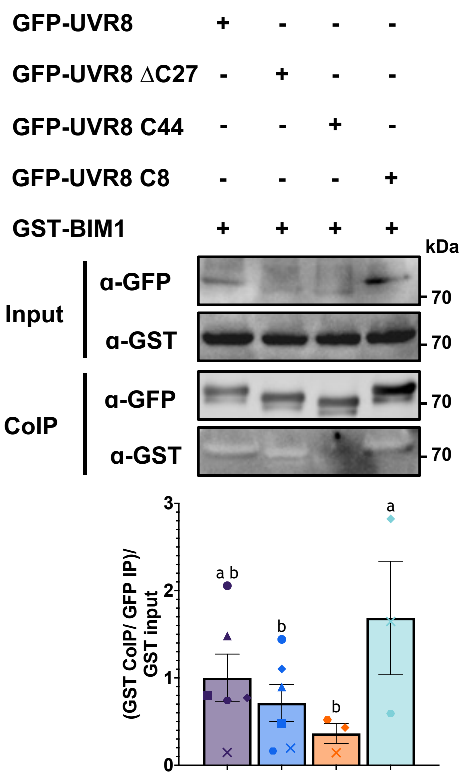

Figure S4 BIM1 interaction with UVR8 in mammalian cells requires the C44 region. GFP-UVR8 (WT or mutants as indicated) and GST-BIM1 were transiently expressed in HEK 293T cells. After 48 hours the cells were exposed to UV-B for 4 hours. Relative expression (Input) was assayed via western blot using anti-GFP (α-GFP) and anti-GST (α-GST) antibodies. GFP-UVR8 was immunoprecipitated from the extract and the amounts of immunoprecipitated GFP-UVR8 (CoIP, α-GFP) and co-immunoprecipitated GST-BIM1 (CoIP, α-GST) were assayed. The upper section of each panel shows representative western blot images and the lower section shows quantification of multiple repeats of each experiment; the bars in each graph correspond to the lanes above. For quantification, the intensity of the GST-BIM1 CoIP band was divided by that of the corresponding GFP-UVR8 IP band and GST-BIM1 Input band. Each data point was normalised to the mean intensity value for WT GFP-UVR8, set to 1. In each graph data points sharing the same symbol were obtained in the same experiment. The data was analysed using a mixed-effects analysis with Tukey's multiple comparison tests. Data are shown ± S.E. Data points significantly different from each other ($p<0.05$) are indicated by different letters above the bars. GFP-UVR8, GFP-UVR8 ∆C27 n=6, GFP-UVR8 C17, GFP-UVR8 C44 n=3. *, $p<0.05$.

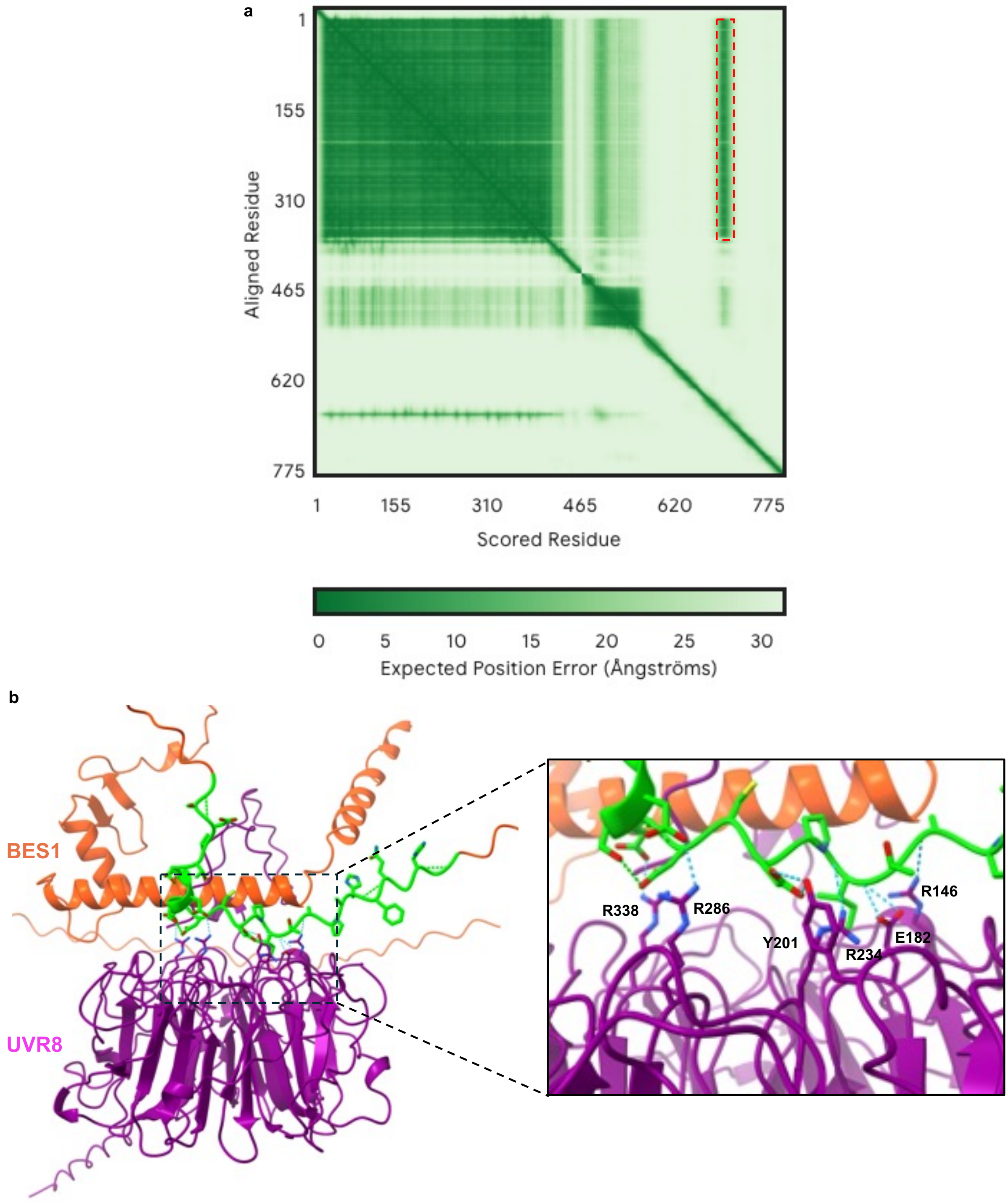

Figure S5 Alphafold analysis of the UVR8-BES1 interaction. The amino acid sequences of UVR8 and BES1 were fed to Alphafold 3 to obtain a predicted structure of the complex of the two proteins from which to derive potential interaction sites and interacting residues to be tested *in vivo.* The programme produced 5 models of the complex's structure. Here we show the results of the first of these models. (a) Predicted alignment error (PAE), also known as Expected Position Error, plot for the

model. This plots a score given by the program in Ångströms (Å) to each residue in both protein sequences relative to every other residue in both proteins that indicates how confident the model is of the predicted position of any one residue with respect to the rest of the structure. Lower scores indicate a more confident prediction. The red dashed box indicates the region of BES1 whose position was most confidently predicted relative to UVR8. We focused on this region to find potential residues within UVR8 that could be important in the interaction with BES1. (b) Structure prediction of the complex. On the left side of the panel is the predicted structure of the UVR8-BES1 complex in its entirety shown in cartoon form. The two proteins are shown in different colours as indicated. The green section indicates the region highlighted by the box in (a). On the right side of the panel is a zoomed-in view of the interface between the two proteins with the residues that could form potential H-bonds between the proteins labelled and shown in stick form (N atoms shown in blue and O atoms shown in red). The H-bonds are shown by blue dashed lines.

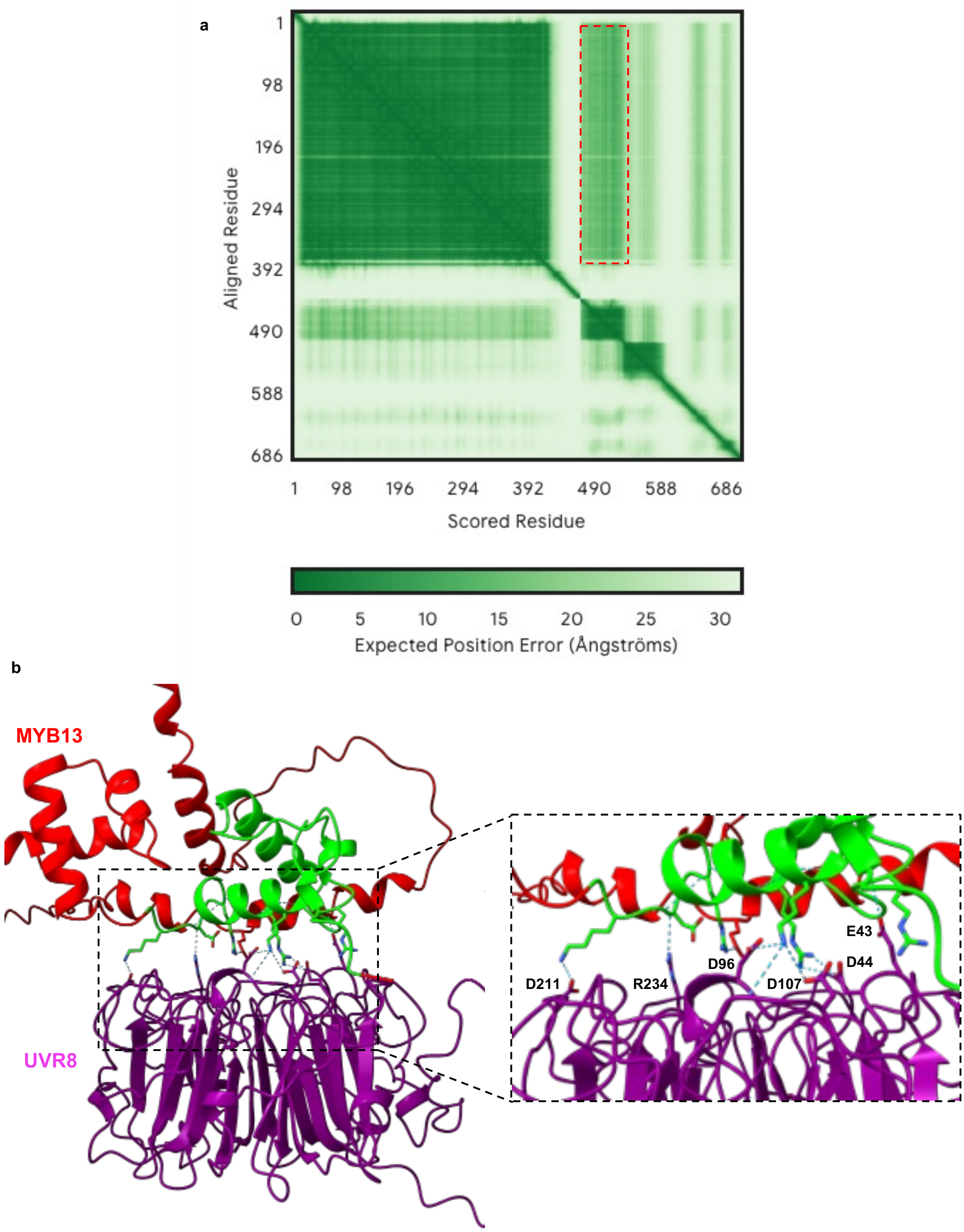

Figure S6 Alphafold analysis of the UVR8-MYB13 interaction. The amino acid sequences of UVR8 and MYB13 were fed to Alphafold 3 to obtain a predicted structure of the complex of the two proteins from which to derive potential interaction sites and interacting residues to be tested *in vivo.* The programme produced 5 models of the

complex's structure. Here we show the results of the first of these models. (a) Predicted alignment error (PAE), also known as Expected Position Error, plot for the model. This plots a score given by the program in Ångströms (Å) to each residue in both protein sequences relative to every other residue in both proteins that indicates how confident the model is of the predicted position of any one residue with respect to the rest of the structure. Lower scores indicate a more confident prediction. The red dashed box indicates the region of MYB13 whose position was most confidently predicted relative to UVR8. We focused on this region to find potential residues within UVR8 that could be important in the interaction with WRKY36. (b) Structure prediction of the complex. On the left side of the panel is the predicted structure of the UVR8-MYB13 complex in its entirety shown in cartoon form. The two proteins are shown in different colours as indicated. The green section indicates the region highlighted by the box in (a). On the right side of the panel is a zoomed-in view of the interface between the two proteins with the residues that could form potential H-bonds between the proteins labelled and shown in stick form (N atoms shown in blue and O atoms shown in red). The H-bonds are shown by blue dashed lines.
